# The Role of Parathyroid Hormone and Vitamin D Serum Concentrations in Patients with Cardiovascular Diseases

**DOI:** 10.1155/2018/5287573

**Published:** 2018-01-31

**Authors:** Agnieszka Kolaszko, Ewa Nowalany-Kozielska, Piotr Ceranowicz, Beata Morawiec, Grzegorz Kubiak

**Affiliations:** ^1^2nd Department of Cardiology, Medical Faculty, Medical University of Silesia, Katowice 10 M. Skłodowskiej-Curie Street, 41-800 Zabrze, Poland; ^2^Department of Physiology, Medical Faculty, Jagiellonian University Medical College, 16 Grzegorzecka Street, 31-531 Krakow, Poland

## Abstract

25-hydroxyvitamin D (25(OH)D) plays a crucial role in human homeostasis. Its deficiency (vitamin D deficiency—VDD), being common in European population, combined with elevated concentration of parathyroid hormone (PTH), represents a vicious cycle of mechanisms leading to heart failure (HF). Despite several papers published in that field, the effect of VDD and PTH concentration on cardiovascular system remains unequivocal; thus, the aim of the study was to compare these data among HF and non-HF patients being prospectively enrolled into the study during hospital stay in the cardiology ward. Patients with HF had higher PTH concentration (85.0 ± 52.6 versus 64.5 ± 31.7, *p* ≤ 0.02) compared to non-HF patients. Mean PTH values were associated with the clinical status expressed by the New York Heart Association class (NYHA class) (“0”—66.04, “I”—56.57, “II”—72.30, “III”—85.59, and “IV”—144.37 pg/ml, *p* ≤ 0.00004). Interestingly, neither 25(OH)D (31.5 versus 29.7 ng/ml, *p* ≤ ns) nor phosphorus (P) (1.23 versus 1.18 mmol/l, *p* ≤ ns) nor total calcium (Ca^2+^) concentration (2.33 versus 2.37 mmol/l, *p* ≤ ns) differed among the groups. Reassuming PTH serum concentration in contrary to 25(OH)D, P and Ca^2+^ are significantly raised among the patients with HF and shows significant relationship with the clinical status expressed by the NYHA class.

## 1. Introduction

A lipid-soluble vitamin D has huge impact on human homeostasis [1]. It is produced during sun exposure and delivered via nutrients (including oily fish and egg yolks), as well as dietary supplements [2]. Vegetable sources provide ergocalciferol (D2), and animal sources provide cholecalciferol (D3). Ergocalciferol and cholecalciferol represent similar metabolic flux being transported to the liver by a vitamin D-binding protein (DBP) and then submitted for hydroxylation at the C25 position by specific hydroxylase. 25-hydroxyvitamin D is the main circulating form of vitamin D [3]. Low serum 25-hydroxyvitamin D (25(OH)D) concentrations are a predominant cause of a negative calcium balance and secondary hyperparathyroidism (SHPT).

SHPT is a frequently occurring entity in patients suffering from heart failure (HF). Since parathormone (PTH) is an important regulator of bone and mineral metabolism, its biological activity is regulated by oscillations in serum calcium concentrations with subsequent reactions on several pathways. The main purpose of those mechanisms is to raise serum concentration of calcium due to enhancement of renal and intestinal reabsorption and osteoclast activation leading to bone resorption. PTH rise in HF clinical setting is related to the impairment of acid-based homeostasis, diuretic-induced calcium loss, and vitamin D deficiency (VDD). Moreover, increased PTH concentrations are related to the impaired hemodynamic state expressed by reduced stroke volume and increased pulmonary capillary wedge pressure being commonly observed in HF patients during right heart catheterization [4].

It is worth to emphasize that 1,25-dihydroxyvitamin D and 25-hydroxyvitamin D have, beyond their important role in calcium and phosphorus metabolism, diverse effects on the immune system [5]. Vitamin D has been reported to protect tissue from myocardial and cerebral ischemia [6, 7]. Many evidence suggests a critical role of vitamin D in blood pressure (BP) regulation; 25(OH)D deficiency stimulates renin-angiotensin-aldosterone system (RAAS) and promotes hypertension [8]. Worldwide studies have shown an association between VDD and tissue inflammation, endothelial dysfunction, arterial stiffness, atherosclerotic plaque formation (coronary heart disease [CAD], peripheral arterial disease), left ventricular hypertrophy, atrial fibrillation, metabolic syndrome, and diabetes. VDD is also associated with an increased risk of death, heart failure, and myocardial infarction (MI) in postmenopausal women, as well as with an increased risk of stroke, MI, and sudden cardiac death or/and death related to other heart diseases among diabetic patients with chronic kidney disease (CKD) [9–18].

VDD is common in European population due to indoor lifestyle and sun avoidance. In Central Europe (CE) effective vitamin D synthesis occurs from April to October only if at least twenty percent of skin is exposed to sunlight for the minimal time of fifteen minutes daily. Sunscreens limit the synthesis in 90%; additionally, VDD is frequently related to smoking, obesity, renal and liver failure, inappropriate eating habits, and drug intake. VDD in obese patients may result from low outdoor activity, inappropriate eating habits, and sequestration of fat-soluble cholecalciferol in adipose tissue [1, 19–22].

VDD combined with raised PTH represents a vicious cycle of mechanisms on one hand leading to HF and in the other hand worsening the prognosis of HF. It is worth to mention that myocardial fibrosis and hypertrophy, excessive adrenergic stimulation, calcium cell overload, oxidative stress enhancement, and FGF-23 formation have huge impact on impaired survival [23].

## 2. Aim of the Study

The aim of the study is to assess and compare the serum intact PTH and 25(OH)D in HF and non-HF patients being prospectively enrolled into the study during hospital stay in the cardiology ward.

## 3. Material and Methods

### 3.1. General Information

This cross-sectional study was designed to asses serum intact PTH, 25(OH)D, total calcium (Ca^2+^), and phosphorus (P) concentrations in addition to routine laboratory examinations and medical history obtained on admission in 140 consecutive patients hospitalized in a clinical ward of cardiology form November 15 to December 31, 2014. The study complies with the Declaration of Helsinki and was approved by the local ethics committee (number of approval KNW/0022/KB1/40/I/12/13), and all patients gave informed consent prior to enrollment.

### 3.2. Inclusion and Exclusion Criteria

Inclusion criteria were as follows: impaired systolic function with left ventricular ejection fraction (LVEF) below 50% according to the Simpson method in the echocardiographic examination or patient diagnosed and/or treated due to HF prior to admission, CAD diagnosed on the base of invasive/noninvasive testing performed before admission, or if patient was previously submitted for coronary intervention. Exclusion criteria were as follows: lack of consent, under eighteen years of age, vitamin D supplementation, or CKD stage four or higher. Invasive coronary angiography (CAG) was found positive for CAD if at least one coronary artery of at least two millimeters was significantly stenosed.

### 3.3. Group Formation

Finally, 127 patients were enrolled into the study. Demographic and clinical data were recorded on admission to the hospital. Patients were requested to characterize their daily profile of sun exposure during the last four months as “above” or “below” seven hours a week (one hour per day).

Patients were post hoc divided into four groups depending on CAD and/or HF occurrence. HF patients were divided into two groups according to the etiology of HF. Ischemic is defined as impaired systolic function with significant narrowings in coronary tree (IHF) and nonischemic (N-IHF) is defined as impaired systolic function without significant narrowings in coronary tree.

Patients with CAD but without HF were depicted as (CAD N-HF) and formed the third group. Patients not fulfilling inclusion criteria of CAD nor HF were depicted as (N-HF&N-CAD) and referred to the fourth group.

### 3.4. Diagnostic Techniques and Further Definitions

Serum concentrations of intact PTH and 25(OH)D were assessed using the immunoenzymatic assay technique with the MicroVue Intact PTH ELISA and 25(OH)-Vitamin D Xpress ELISA Kit, respectively. Serum P and Ca^2+^ concentration was assessed using the enzymatic method as described by Spinreact S.A., Spain, and the colorimetric Arsenazo III method as described by Biomaxima S.A., Poland.

Arterial hypertension was diagnosed if systolic and diastolic blood pressures exceeded 140 and/or 90 mmHg or if the patient already received antihypertensive treatment. Diabetes was diagnosed according to the American Diabetes Association (ADA) criteria or in patients already receiving antidiabetic medication. The estimated glomerular filtration rate (eGFR) was calculated on the basis of the Modification of Diet in Renal Disease (MDRD) formula.

**Figure 1:**
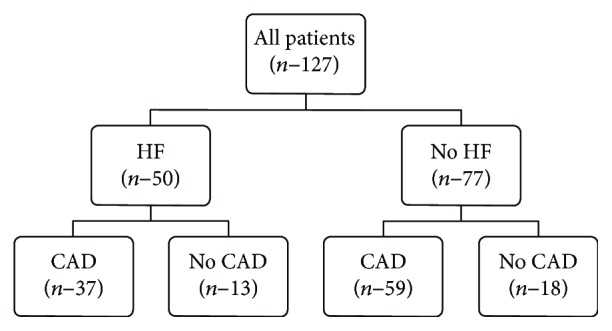
Subgroups of patients. HF: heart failure; No HF: heart failure absent; CAD: coronary artery disease present; No CAD: coronary artery disease absent.

For the design of the study and patient characteristic, please refer to Figure 1 and Table 1.

## 4. Statistical Analysis

Distributions of the examined parameters were analyzed using the Shapiro-Wilk test. Values were presented as the mean and standard deviation (SD) or as the median in the 25th and 75th percentiles. Nominal and categorical values were expressed in percentages or proportional rates. Linear variables with normal distribution were compared using student *t*-test. Post hoc analyses between the groups were performed by the use of factorial ANOVA test with the Bonferroni correction. Variables with abnormal distribution were compared using the Mann–Whitney *U* test. Categorical variables of abnormal distribution were compared using Chi-squared test with Yates correction. Quantified Spearman's rho correlation coefficients were used to assess the linear correlations between LVEF and PTH among all specified groups. Differences between the values were considered statistically significant if *P* ≤ 0.05. Analyses were performed using Statistica 10 with medical package (Statsoft Inc.).

## 5. Results

Patients with HF had higher incidence of atrial fibrillation (AF) and were characterized by significant negative remodeling of the left ventricle expressed by lower LVEF and higher end-diastolic volume (EDV). Incidence of implantable cardioverter-defibrillator (ICD) was significantly elevated in the HF group and reached borderline significance as far as frequency of dual chamber permanent pacemaker (DDDR) was concerned. The remaining parameters depicted in Table 1 did not show statistically significant differences.

Patients with HF had deteriorated renal function expressed by significantly increased serum creatinine concentration and dropped eGFR of borderline statistical significance. PTH in HF patients was significantly elevated. There was no incidence of PTH-dependent hypercalcemia in examined group of patients. The rest of the findings are depicted in Table 2 and did not show statistical significance.

**Figure 2:**
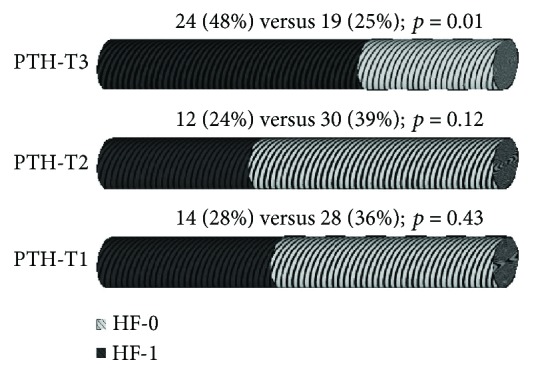
Incidence of HF divided according to tertiles of PTH serum concentration. HF: heart failure; PTH: parathyroid hormone; T: tertile.

The number of patients with the PTH serum concentration values in the third tertile was significantly raised among HF versus non-HF patients—data are presented in Figure 2.

Patients in N-IHF group were younger, had lower occurrence rate of hypertension and dyslipidemia, and had significantly decreased platelet blood count compared to IHF group of patients. Patients in CAD N-HF group had decreased white blood count and EDV and increased LVEF compared to IHF group of patients with trend towards significance considering PTH serum blood concentration. Data are depicted in Tables 3 and 4.

N-IHF patients were younger, had significantly decreased LVEF, and had increased EDV compared with N-HF&N-CAD group of patients. Dyslipidemia, AF, and ICD implantation in IHF patients were observed more frequently compared to CAD N-HF group of patients. The remaining parameters did not show statistical significance among the groups—data were presented in Tables 3 and 4.

**Figure 3:**
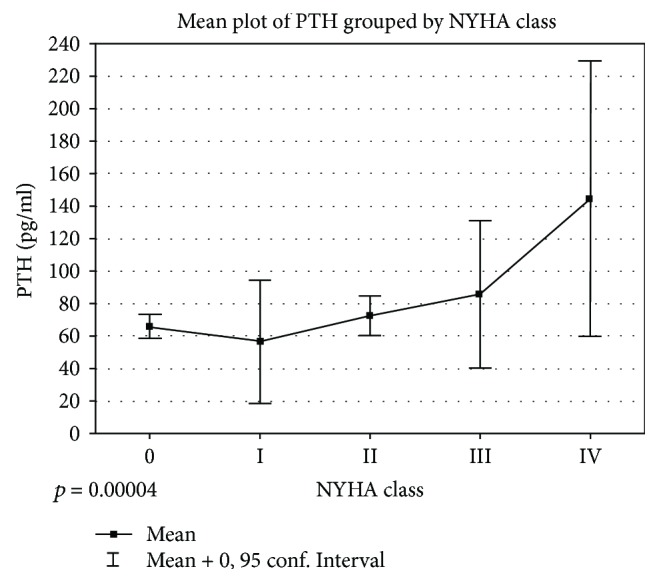
Mean plot of PTH grouped by NYHA class. NYHA: New York Heart Association; PTH: parathyroid hormone.

The mean PTH concentration was significantly increased in patients in worse clinical state expressed by the NYHA class; results were reported in Figure 3 and in Table 5.

PTH and LVEF showed statistically significant correlation in all patients, in HF and in IHF patients. In N-IHF patients, only borderline significance was reported. There was no significant correlation in patients without HF. Data were presented in Table 6.

PTH concentration showed statistically significant correlation with EDV, LVEF, serum creatinine concentration, eGFR, and N-terminal probrain natriuretic peptide serum concentration (NT-proBNP). Data were presented in Table 7.

PTH concentration was significantly elevated in patients treated with loop diuretics (LD), with the highest values observed in HF patients. Data was presented in Table 8.

Neither PTH nor 25(OH)D concentrations were dependent on self-reported sun exposure in HF and N-HF patients.

## 6. Discussion

The presented study, according to our best knowledge, is the first to compare vitamin D, PTH, calcium, and phosphorus serum concentration in cardiology ward patients with and without HF taking into account its etiology (ischemic versus nonischemic). There were no differences in vitamin D concentration among the groups although it oscillated between lower normal limits.

Self-reported sunlight exposure is a novel approach. The cut-off value was set up on one hour per day what we believe provides optimal vitamin D synthesis.

Patients with HF had higher rates of PTH, irrespectively of its etiology what remains in line with previously published studies [23–27]. PTH concentration in HF correlates with disease severity, expressed by decreased LVEF, increased EDV, and functional NYHA class. It also correlates with lower eGFR and higher NT-proBNP concentration what is coherent with previous reports [28–31].

SHPT, being an exclusive cause of elevated PTH concentration in our study, is a common state in HF patients originating from several overlapping mechanisms. Increased aldosterone activity (compensatory activation of RAAS) and loop diuretic intake promote excessive urine calcium and magnesium loss. Renal dysfunction, due to chronic hypoperfusion, increases P retention and 25(OH)D activation disturbance (decreased activity of renal 1-alpha-hydroxylase induced by uremia), which stimulates PTH secretion. This is coherent with our findings: LD intake affected PTH concentration in all participants; its predominant influence was observed in HF patients.

25(OH)D insufficiency, common in HF, is also a cause of SHPT [29, 30]. There are reports showing independent association between higher PTH level and an exacerbated risk of HF in the general population [28]. SHPT increases bone turnover and the risk of fractures; it is also associated with cardiovascular calcifications and higher mortality risk. Treatment of severe SHPT in CKD is based on control of dietary phosphate intake, gastrointestinal absorption (chelating agents), vitamin D supplementation, and calcimimetic drug intake [32]. It is not fully elucidated whether treatment of SHPT in HF patients has beneficial effect on prognosis; thus, we think further studies are required in this field.

Polish Society of Endocrinology recommends at least 15 minutes of sun exposure of 15–20% of body surface daily, from April to October, in order to provide optimal 25(OH)D serum concentration [33]. In our analysis, the assumed criterion of optimal-declared sun exposure time was above 7 hours per week during the last 4 months, which was equivalent of one hour per day and outstrips common recommendations. The study was performed during November and December; thus, 25(OH)D concentration to a lesser degree depended on endogenous 25(OH)D synthesis.

There were no differences between examined groups in self-reported sunlight exposure; it also had no influence on vitamin D or PTH concentration. Against the odds, HF patients did not declare decreased sun exposure in comparison to the rest of the cardiology ward patients. Moreover, self-reported sunlight exposition did not affect 25(OH)D serum concentration: in group of higher sun exposure, 25(OH)D serum concentration was 30.73 ng/ml versus 29.89 ng/ml (*p* = ns). Partially, it could have resulted from the lack of skin synthesis in months preceding blood sampling, from a relatively small number of study participants or from patients' personal fitness overrating and disease negation.

Decreased serum 25(OH)D concentration in HF patients, correlating with disease severity, was reported by numerous authors [34]. In our research, mean 25(OH)D concentration did not differ among the groups which corroborates with our previous findings [31].

CE population, with regard to latitude, is prone to 25(OH)D deficiency especially during winter. Despite the fact that blood samples were collected in November and December, mean 25(OH)D concentration oscillated between normal limits in both groups. 25(OH)D deficiency requiring treatment (<20 ng/ml) occurred in 30 patients (23.6%), and suboptimal concentration (20–30 ng/ml) was stated in 24 participants (18.9%). The range of 25(OH)D concentration was 12.0–46.6 ng/ml in all patients. Recent large cross-sectional study reported by Płudowski et al. showed considerable prevalence of 25(OH)D deficiency in Polish volunteers, with mean concentration amounting 18.0 ± 9.6 ng/ml; deficiency (<20 ng/ml) occurred in 65.8% participants, and suboptimal concentration (20–30 ng/ml) was stated in 24.1%. Sampling was performed in March and May and mean 25(OH)D concentration in late winter was 17.7 ± 10.1 ng/ml, in spring 18.3 0 ± 9.1 ng/ml, respectively [35]. Discrepancies between these values were most probably caused by different time of blood sampling, considering the half-life of 25(OH)D defined was twenty-five days, and lack of skin synthesis in winter could influence the findings. This so-called “seasonal effect” being coherent with our results was also reported by Tokarz et al. in the acute myocardial infarction group of patients [36]. It is worth to mention that 25(OH)D concentration during winter is based upon food intake and previous liver storage. Comparing to Płudowski et al. [35], our patients were older (63.8 versus 54.0 years), had higher BMI (28.3 versus 26.0), and were mostly men (62.2% versus 22.7%). Moreover, measurements were performed with laboratory assays provided by different companies. The problem of 25(OH)D standardization had been brought up in previously published studies. Immunoassays, being sensitive to 24,25-dihydroxyvitamin D, can overrate the serum 25(OH)D concentrations up to 5 ng/ml [37–40].

On the other hand, Gruson et al. showed that 1,25(OH)2D, but not 25(OH)D, correlates with NYHA functional class in chronic heart failure patients, especially that 1,25(OH)2D to PTH(1–84) ratio is a promising predictor of cardiovascular death in HF [41, 42]. There are some other reports showing that 25(OH)D deficiency was not related to HF in contrast to PTH [43, 44]. Being aware of limitations, we decided to asses 25(OH)D in our study because of easier access and lower costs than 1,25(OH)D assessment, as well as the wide range of other studies comparing 25(OH)D concentration in circulatory system.

Patients with AF had higher PTH concentration. In HF group, AF occurrence was more frequent, what is related to adverse remodeling of the myocardium but also may be affected by elevated PTH concentration, which is a proarrhythmic agent. An opposite causal link is also presumable: AF may promote PTH elevation [45]. In CAD group, higher number of white blood cells was observed when compared to patients without ischemia, with the highest values in IHF. It could be explained by the most excessive inflammatory state in advanced atherosclerosis, complicated by heart muscle damage. Statistically, significant difference between PLT count in IHF versus N-IHF (215.78 versus 169.62, *p* ≤ 0.04) group of patients is related with unfavorable prognosis and was widely reported in the literature [46–48].

## 7. Limitations of the Study

The results of the study might have been biased on several pathways. The main limitation of this study is its cross-sectional design, self-reported data about sun exposure, and relatively small amount of patients; however, the study had a strictly pilot character. The researchers did not interfere with the management process; nevertheless, the data were acquired from a single cardiology center providing management to the population of approximately one million inhabitants.

## 8. Conclusions

PTH serum concentration is significantly raised in patients with HF irrespectively of its etiology and correlates with LVEF, NYHA functional class, renal function, and loop diuretics intake. Vitamin D serum concentration oscillates between lower normal limit in all patients and is not affected by the HF, CAD occurrence, or declared exposure to sun radiation.

## Figures and Tables

**Table 1:** Patient characteristic divided depending on heart failure occurrence.

	HF present (*n*–50)	HF absent (*n*–77)	*p*
Age	61.5 (57.0–74.0)	65.3 (57.0–74.0)	ns
Male (%)	36 (72)	43 (56)	ns
BMI	28.1 (24.3–30.9)	28.5 (25.4–31.2)	ns
Hypertension	38 (76)	61 (79)	ns
Dyslipidemia	32 (64)	38 (49)	ns
Diabetes	15 (30)	27 (35)	ns
Atrial fibrillation	14 (28)	8 (10)	≤0.02
Urgent admission	22 (44)	34 (44)	ns
Sun exposure >7 h/week	26 (52)	50 (65)	ns
Coronary artery disease	39 (78)	59 (77)	ns
EDV (ml)	146.2 ± 55.9	86.3 ± 20.9	≤0.001
LVEF (%)	28.7 ± 11.7	55.9 ± 4.7	≤0.001
ICD	12 (24)	1 (1)	≤0.001
CRT	2 (4)	0 (0)	ns
DDDR	2 (4)	13 (17)	≤0.06
VVIR	2 (4)	5 (5)	ns
OHT	2 (4)	0 (0)	ns
DEATH (during hospitalization)	2 (4)	2 (3)	ns

HF: heart failure; BMI: body mass index; >7 h/week: more than seven hours per week; EDV: end-diastolic volume; LVEF: left ventricular ejection fraction; ICD: implantable cardioverter-defibrillator; CRT: cardiac resynchronization therapy; DDDR: dual chamber permanent pacemaker; VVIR: single chamber permanent pacemaker; OHT: orthotopic heart transplantation.

**Table 2:** Clinical and laboratory findings depending on heart failure occurrence.

	HF present (*n*–50)	HF absent (*n*–77)	*p*
eGFR (ml/min/1.73^2^)	76.1 ± 26.1	82.9 ± 22.6	≤0.09
Creatinine (mmol/l)	90.0 ± 30.9	81.1 ± 48.5	≤0.01
ALT (IU/l)	30.5 ± 21.4	31.2 ± 22.6	ns
AST (IU/l)	35.5 ± 34.2	27.1 ± 14.3	ns
HGB (g/dl)	13.5 ± 1.5	13.6 ± 1.5	ns
HCT (%)	41.3 ± 6.9	41.8 ± 6.0	ns
RBC (10^6^/ul)	4.47 ± 0.6	4.46 ± 0.5	ns
PLT (10^3^/ul)	203.8 ± 66.6	210.0 ± 53.8	ns
WBC (10^3^/ul)	7.5 ± 2.6	6.8 ± 2.2	ns
25(OH)D (ng/ml)	31.5 ± 8.94	29.7 ± 10.2	ns
Ca^2+^ (mmol/l)	2.33 ± 0.13	2.37 ± 0.1	ns
P (mmol/l)	1.23 ± 0.17	1.18 ± 0.2	ns
PTH (pg/ml)	85.0 ± 52.6	64.5 ± 31.7	≤0.02

HF: heart failure; eGFR: estimated glomerular filtration rate; ALT: alanine aminotransferase; AST: aspartate aminotransferase; HGB: hemoglobin; HCT: hematocrit; RBC: red blood cells; PLT: platelets; WBC: white blood cells; 25(OH)D: 25-hydroxyvitamin D; Ca^2+^: total calcium; P: phosphorus; PTH: parathyroid hormone.

**Table 3:** Laboratory findings among all examined groups.

		HF			CAD			No CAD	
	N-IHF *n*–13	IHF *n*–37		IHF *n*–37	CAD N-HF *n*–59		N-IHF *n*–13	N-HF & N-CAD *n*–18	
Parameter	Versus		*p*	Versus		*p*	Versus		*p*
25(OH)D (ng/ml)	29.25	32.29	ns	32.29	29.47	ns	29.25	31.00	ns
Ca^2+^ (mmol/l)	2.36	2.32	ns	2.32	2.37	ns	2.36	2.37	ns
P (mmol/l)	1.21	1.23	ns	1.23	1.18	ns	1.21	1.18	ns
PTH (pg/ml)	85.26	84.91	ns	84.91	64.24	≤0.06	85.26	62.67	ns
eGFR (ml/min/1.73^2^)	67.38	79.14	ns	79.14	81.15	ns	67.38	87.88	ns
Creatinine (mmol/l)	96.00	87.89	ns	87.89	84.11	ns	96.00	71.06	ns
ALT (IU/l)	35.23	28.84	ns	28.84	29.73	ns	35.23	37.47	ns
AST (IU/l)	38.69	34.41	ns	34.41	26.32	ns	38.69	30.53	ns
HGB (g/dl)	13.52	13.48	ns	13.48	13.51	ns	13.52	13.80	ns
HCT (%)	42.44	40.86	ns	40.86	41.36	ns	42.44	42.97	ns
RBC (10^6^/ul)	4.61	4.42	ns	4.42	4.43	ns	4.61	4.53	ns
PLT (10^3^/ul)	169.62	215.78	≤0.04	215.78	212.29	ns	169.62	202.88	ns
WBC (10^3^/ul)	6.68	7.79	ns	7.79	7.04	≤0.04	6.68	5.87	ns

N-IHF: nonischemic heart failure; IHF: ischemic heart failure; CAD N-HF: coronary artery disease without heart failure; N-HF&N-CAD: without coronary artery disease nor heart failure; 25(OH)D: 25-hydroxyvitamin D; Ca^2+^: total calcium; P: phosphorus; PTH: parathyroid hormone; eGFR: estimated glomerular filtration rate; ALT: alanine aminotransferase; AST: aspartate aminotransferase; HGB: hemoglobin; HCT: hematocrit; RBC: red blood cells; PLT: platelets; WBC: white blood cells.

**Table 4:** Clinical findings among all examined groups.

		HF			CAD			No CAD	
*n* (%)	N-IHF *n*–13	IHF *n*–37		IHF *n*–37	CAD N-HF *n*–59		N-IHF *n*–13	N-HF & N-CAD *n*–18	
Parameter	Versus		*p*	Versus		*p*	Versus		*p*
Age (years)	51.54	65.00	≤0.001	65.00	66.37	ns	51.54	62.82	≤0.03
BMI	25.83	28.91	ns	28.91	28.43	ns	25.83	29.02	ns
EDV (ml)	163.08	140.32	ns	140.32	87.03	≤0.001	163.08	84.82	≤0.001
LVEF (%)	24.62	30.11	ns	30.11	55.25	≤0.001	24.62	58.53	≤0.001
Male	8 (62)	28 (76)	ns	28 (76)	34 (58)	ns	8 (62)	8 (47)	ns
HT	5 (38)	33 (89)	≤0.001	33 (89)	49 (83)	ns	5 (38)	12 (71)	ns
DYSLIP	4 (31)	28 (76)	≤0.01	28 (76)	31 (53)	≤0.04	4 (31)	7 (41)	ns
Diabetes	3 (23)	12 (32)	ns	12 (32)	24 (41)	ns	3 (23)	3 (18)	ns
AF	4 (31)	10 (28)	ns	10 (28)	5 (8)	≤0.03	4 (31)	3 (18)	ns
Urgent	4 (31)	18 (49)	ns	18 (49)	28 (47)	ns	4 (31)	6 (35)	ns
SE > 7 h/w	7 (54)	19 (51)	ns	19 (51)	41 (69)	ns	7 (54)	9 (53)	ns
ICD	5 (38)	7 (19)	ns	7 (19)	0 (0)	≤0.001	5 (38)	1 (6)	ns
CRT	1 (8)	1 (3)	ns	1 (3)	0 (0)	ns	1 (8)	0 (0)	ns
DDDR	0 (0)	2 (5)	ns	2 (5)	8 (14)	ns	0 (0)	5 (29)	ns
VVIR	1 (8)	1 (3)	ns	1 (3)	2 (3)	ns	1 (8)	2 (12)	ns
OHT	2 (15)	0 (0)	ns	0 (0)	0 (0)	ns	2 (15)	0 (0)	ns
Death	0 (0)	2 (5)	ns	2 (5)	2 (3)	ns	0 (0)	0 (0)	ns

N-IHF: nonischemic heart failure; IHF: ischemic heart failure; CAD N-HF: coronary artery disease without heart failure; N-HF&N-CAD: without coronary artery disease nor heart failure; BMI: body mass index; EDV: end-diastolic volume; LVEF: left ventricular ejection fraction; HT: hypertension; DYSLIP: dyslipidemia; AF: atrial fibrillation; urgent: urgent admission; SE > 7 h/week: sun exposure exceeding seven hours per week; ICD: implantable cardioverter-defibrillator; CRT: cardiac resynchronization therapy; DDDR: dual chamber permanent pacemaker; VVIR: single chamber permanent pacemaker; OHT: orthotopic heart transplantation.

**Table 5:** PTH values (pg/ml) divided according to NYHA class.

NYHA	*N*	Mean	Median	Lower quartile	Upper quartile	SD	*p*
All	127	72.54	60.93	42.61	88.80	42.26	0.00004^∗∗^
0	73	66.04	57.49	42.61	82.24	31.29	0.000001^∗^
I	7	56.57	47.11	25.00	65.81	41.06	0.000043^∗^
II	33	72.30	72.01	48.55	89.97	34.15	0.000017^∗^
III	7	85.59	89.86	38.44	112.93	49.19	0.005274^∗^
IV	7	144.37	117.97	62.70	203.98	91.66	—

^∗∗^All effects tested by ANOVA; ^∗^Post hoc comparison versus NYHA IV by the use of Fischer LSD test. NYHA: New York Heart Association; SD: standard deviation.

**Table 6:** Correlation between PTH concentration and LVEF in different groups of patients.

Group of patients	Spearman Rho coefficients	*p*
All (*n*–127)	−0.3479	≤0.00006
HF (*n*–50)	−0.4168	≤0.0026
N-HF (*n*–77)	0.0480	ns
N-IHF (*n*–13)	−0.5409	≤0.0563
IHF (*5*)	−0.3883	≤0.0176

HF: heart failure; N-HF: without heart failure; N-IHF: nonischemic heart failure; IHF: ischemic heart failure.

**Table 7:** Correlation between PTH and subsequent parameters.

All patients	Spearman Rho coefficients	*p*
EDV	0.269	0.002205
LVEF	−0.290	0.000953
eGFR	−0.375	0.000014
creatinine	0.407	0.000002
NT-proBNP	0.449	0.016441

EDV: end diastolic volume; LVEF: left ventricle ejection fraction; eGFR: estimated glomerular filtration rate; NT-proBNP: N-terminal probrain natriuretic peptide.

**Table 8:** PTH concentration (pg/ml, mean ± SD) according to loop diuretic intake and HF occurrence.

Loop diuretic intake	(+)	(−)	*p*
All (*n* = 127)	(*n* = 46) 99.09 ± 53.78	(*n* = 81) 58.26 ± 26.89	0.00000015

HF (*n* = 50)	(*n* = 28) 105.77 ± 58.45	(*n* = 22) 60.15 ± 30.49	0.000452

N-HF (*n* = 77)	(*n* = 14) 84.06 ± 39.48	(*n* = 63) 57.63 ± 25.87	0.008666

HF patients: heart failure patients; N-HF patients: patients without heart failure. Loop diuretics: furosemide and torasemide.
