# Corrigendum to “The Role of Parathyroid Hormone and Vitamin D Serum Concentrations in Patients with Cardiovascular Diseases”

**DOI:** 10.1155/2018/2046808

**Published:** 2018-12-02

**Authors:** Agnieszka Kolaszko, Ewa Nowalany-Kozielska, Piotr Ceranowicz, Beata Morawiec, Grzegorz Kubiak

**Affiliations:** ^1^2nd Department of Cardiology, Medical Faculty, Medical University of Silesia, Katowice 10 M. Skłodowskiej-Curie Street, 41-800 Zabrze, Poland; ^2^Department of Physiology, Medical Faculty, Jagiellonian University Medical College, 16 Grzegorzecka Street, 31-531 Krakow, Poland

In the article titled “The Role of Parathyroid Hormone and Vitamin D Serum Concentrations in Patients with Cardiovascular Diseases” [1], the “≤” signs should be replaced with “=” signs throughout the article. This correction has been made in place.
